# Evaluating the Effect of Thermal Treatment on Phenolic Compounds in Functional Flours Using Vis–NIR–SWIR Spectroscopy: A Machine Learning Approach

**DOI:** 10.3390/foods14152663

**Published:** 2025-07-29

**Authors:** Achilleas Panagiotis Zalidis, Nikolaos Tsakiridis, George Zalidis, Ioannis Mourtzinos, Konstantinos Gkatzionis

**Affiliations:** 1 Laboratory of Consumer and Sensory Perception of Food & Drinks, Department of Food Science and Nutrition, University of the Aegean, Metropolite Ioakeim 2, 81400 Myrina, Greece; fnsd21005@aegean.gr; 2 Laboratory of Remote Sensing, Spectroscopy and Geographic Information Systems (GIS), School of Agriculture, Aristotle University of Thessaloniki, 54124 Thessaloniki, Greece; tsakirin@auth.gr; 3 Laboratory of Food Chemistry and Biochemistry, Department of Food Science and Technology, School of Agriculture, Aristotle University of Thessaloniki, 54124 Thessaloniki, Greece; mourtzinos@agro.auth.gr

**Keywords:** near infrared spectroscopy, stability of phenolics, thermal treatment, functional flours, olive-by-products, pulse flours, artificial intelligence

## Abstract

Functional flours, high in bioactive compounds, have garnered increasing attention, driven by consumer demand for alternative ingredients and the nutritional limitations of wheat flour. This study explores the thermal stability of phenolic compounds in various functional flours using visible, near and shortwave-infrared (Vis–NIR–SWIR) spectroscopy (350–2500 nm), integrated with machine learning (ML) algorithms. Random Forest models were employed to classify samples based on flour type, baking temperature, and phenolic concentration. The full spectral range yielded high classification accuracy (0.98, 0.98, and 0.99, respectively), and an explainability framework revealed the wavelengths most relevant for each class. To address concerns regarding color as a confounding factor, a targeted spectral refinement was implemented by sequentially excluding the visible region. Models trained on the 1000–2500 nm and 1400–2500 nm ranges showed minor reductions in accuracy, suggesting that classification is not solely driven by visible characteristics. Results indicated that legume and wheat flours retain higher total phenolic content (TPC) under mild thermal conditions, whereas grape seed flour (GSF) and olive stone flour (OSF) exhibited notable thermal stability of TPC even at elevated temperatures. These first findings suggest that the proposed non-destructive spectroscopic approach enables rapid classification and quality assessment of functional flours, supporting future applications in precision food formulation and quality control.

## 1. Introduction

In recent years, there has been a growing interest in employing ingredients for fortifying food and enriching its health promoting properties [1]. Functional flours, derived from a variety of grains, seeds, or legumes, have gained popularity due to their diverse health benefits, such as reducing the risk of chronic diseases and promoting overall well-being [2,3]. In contrast to wheat flour, which lacks desirable nutritional attributes [4], these flours are rich in bioactive compounds, including phenolic compounds, which exhibit antioxidant, anti-inflammatory, and antimicrobial properties [5]. Pulse flours, such as lupin flour and chickpea flour, are derived from legumes and considered to be rich sources of phenolic compounds [6]. Additionally, there is a growing interest in utilizing plant-based ingredients that are rich in phytochemicals, which would otherwise end up as waste [7]. These ingredients include grape pomace, which can be processed into grape seed flour, and olive stones, which can be ground into fine olive stone powder or flour.

Different types of functional flours are characterized by distinct profiles of phenolic compounds. For instance, wheat flour predominantly contains ferulic acid, vanillic acid, and p-coumaric acid, primarily in bound forms [8]. Lupin flour is rich in flavonoids and phenolic acids such as caffeic and trans-ferulic acid, which contribute to its antioxidant potential [9]. Chickpea flour contains notable amounts of isoflavones and phenolic acids including gallic and protocatechuic acid [10]. Grape seed flour is especially rich in flavan-3-ols like catechin and epicatechin, as well as proanthocyanidins while olive stone flour typically contains tyrosol and oleuropein which are potent antioxidants [11]. These distinct phenolic profiles not only influence the health-promoting properties of each flour but also affect their stability during processing, underscoring the importance of characterizing their behavior under thermal treatment.

Incorporating functional flours into food formulations presents a chance to enhance their nutritional profiles and improve product functionality [12]. Mild thermal treatment [13] of flours has been previously associated with the improvement of shelf-life [14] and microbial stability [15]. However, heat treatment may affect the stability of phenolic compounds and cause degradation, transformation, or loss of certain properties, thereby affecting the functional attributes of the final products [16]. Understanding the changes in phenolic compound composition induced by thermal treatment is crucial for optimizing processing conditions, selecting the appropriate matrices to develop novel food products and preserving bioactive properties.

Conventional methods for phenolic compounds determination, such as high-performance liquid chromatography (HPLC) and liquid chromatography-mass spectrometry (LC-MS), are well-established and provide precise and reliable results. However, they are time-consuming, labor-intensive, and require the use of expensive reagents. Moreover, they involve complex sample preparation procedures that may cause sample degradation and alter the chemical composition [17].

In recent years, visible, near-infrared and shortwave infrared (Vis–NIR–SWIR) spectroscopy has gained recognition as a rapid and non-destructive analytical tool for quality assessment and compositional analysis of various food products [18,19]. Vis–NIR–SWIR spectroscopy operates in the spectral range of approximately 350–2500 nm and exploits the absorbance properties of chemical components in the sample. Although the fundamental absorption features generated due to molecular motion like translation, rotation and vibrations, may be found in higher wavelengths and specifically in the mid-infrared region, the overtones and combination bands of these features are present in lower wavelengths, enabling material characterization [20]. The technique offers several advantages over traditional methods, including real-time analysis, minimal sample preparation, and the potential for high-throughput screening [21]. However, there are some known limitations: the overlapping spectral features from the overtones and combinations bands make the direct connection between wavelength and specific chemical bond far more involved compared to mid-infrared spectra, requiring the deployment of machine learning algorithms [22]. What is more, the presence of many confounding factors, such as particle size, sample texture [23] and moisture content, dictate that careful protocols should be utilized during the preparation of the sample and the acquisition of its Vis–NIR–SWIR reflectance spectrum.

Previous studies have deployed Vis–NIR–SWIR spectroscopy to evaluate the phenolic content of wheat flour with great accuracy and bioactive compounds in barley flour with ranging accuracy [24,25]. Additionally, the effect of thermal treatment in phenolic compounds has been investigated in black rice flour using fluorescence spectroscopy [26]. A more comprehensive study where various functional matrices are assessed at the same time regarding the stability of phenolic compounds under thermal treatment would be of great value.

Artificial intelligence through machine learning (ML) algorithms has revolutionized the predictive performance of current chemometric methods used in the food sector [27]. Kusumiyati and Asikin (2023) [28] have previously used near-infrared spectroscopy (NIR) along with machine learning models to predict the total phenolic content (TPC) in several horticultural products. In addition, ML techniques were deployed to assess anthocyanin content of onion waste through Vis–NIR–SWIR spectroscopy [29]. In this context, the use of Vis–NIR–SWIR spectrometers coupled with chemometric methods and feature importance analysis [30] could facilitate decision-making at all relevant stages across the flour supply chain. The phenolic content classification along with the identification of flour type and the detection of thermal treatment could provide useful tools for the food industry to select the appropriate matrices when developing novel food products.

The objective of this study was to (i) determine the effect of thermal treatment on certain functional flours commonly used in the food industry and (ii) assess the ability of Vis–NIR–SWIR spectroscopy, a rapid and non-destructive analytical technique, in accurately classifying the flours and determining their phenolic content across thermal treatments. The selection of the functional flours was made based on both compositional and functional considerations. Wheat, lupin and chickpea flour are well-established ingredients in the food industry, commonly used as sources of carbohydrates and plant proteins [31]. Grape seed and olive stone flours represent promising functional ingredients sourced from agri-food waste. Phenolic content, thermal treatment and flour type constituted the classification parameters. Reference (TPC) measurements were obtained using Folin–Ciocalteu assay and the flour samples were thermally treated in various temperatures to evaluate thermal stability of the bioactive compounds. The corresponding spectral signatures were recorded under standard acquisition protocols and a series of widely used baseline machine learning algorithms were deployed using the whole Vis–NIR–SWIR spectra as variable input. The main novelty of this work was the ability: (i) to classify functional flours based on their phenolic content in a simple, inexpensive, and non-destructive way, (ii) to discern whether flour has undergone thermal processing and (iii) to access Vis–NIR–SWIR spectroscopy as an operational tool for future classification of flours.

## 2. Materials and Methods

### 2.1. Flour Samples and Chemicals

Flours used in the study were sourced from commercial retailers and included: wheat flour (WF) from Miloi Agiou Georgiou in Keratsini, Greece; lupin flour (LF) from Lup’Ingredients in Martigne-Ferchaud, France; chickpea flour (CF) from Bioagros in Pella, Greece; grape seed flour (GSF) from PaleoCentrum in Budapest, Hungary, and olive stone flour (OSF) from Nutexa in Valencia, Spain.

Large 10 kg sacks of each flour were first divided into 3 kg working batches. From each batch, 500 g was collected and thermally treated at five different temperatures (25, 74, 110, 145, and 180 °C). For each flour type, twelve samples of 40 g were prepared, yielding a total of 300 unique physical samples (5 flour types × 12 samples × 5 thermal treatments) placed into individual containers. Folin–Ciocalteu reagent and sodium carbonate were obtained from Merck (Darmstradt, Germany). Gallic acid (>99%) was purchased from Sigma Chemical Co. (St. Louis, MO, USA). All solvents and analytical standards were of analytical grade and were purchased from Sigma-Aldrich, Chemie GmbH (Taufkirchen, Germany).

### 2.2. Granulometric Analysis

To assess the particle size distribution of the flour samples granulometric analysis was performed using a vibratory sieve shaker (Retax, Labor Siebmaschine, Type LS10, Nr 4082, Haan, Germany) based on the standard AACC method 66-20.01 [32]. Approximately 100 g of each flour sample were sieved through a standardized stack of stainless-steel sieves with mesh sizes of 600, 500, 350, 250, 175, 79.5, and 9 µm for a duration of 15 min at a constant amplitude. The retained weight at each sieve was recorded, and results were expressed as a percentage of the total sample weight (Table S1, Supplementary material).

### 2.3. Chemical Characterization of Flours

#### 2.3.1. Heat Treatment of Flours

The effect of heat treatment on the TPC of flours was investigated by thermal processing of the materials at 74 °C [13] and at temperatures common in baking (110, 145, 180 °C). A control sample was used at 25 °C. Samples of flours (500 g), derived from the larger batches (3 kg), were placed as a single layer of approximately 3 cm in height in aluminum dishes (30 cm diameter), which were then covered with perforated aluminum lids to prevent sample shifting and ensure uniform exposure while allowing moisture release. The dishes were placed in a preheated, fan-assisted hot-air oven and were heated for 30 min. To further reduce excessive moisture loss during treatment, a beaker of distilled water was placed inside the oven to create a mildly humidified atmosphere, helping maintain a consistent moisture environment around the samples. The samples were then cooled at room temperature in a desiccator and were subsequently stored at −20 °C until analysis.

#### 2.3.2. Determination of Total Phenolic Content (TPC)

Sample extraction was achieved by mixing 10 g of each flour obtained from the thermal processing described in Section 2.3.1 with 50 mL of 80:20 MeOH:H_2_O. The solution was mixed by vortexing at 600 rpm for 30 min and then subjected to an ultrasonic bath for another 30 min. The supernatant was collected by syringe and transferred to Falcon tubes and kept refrigerated. The TPC of the flour extracts was determined according to a published protocol [33] using the Folin–Ciocalteu methodology and gallic acid as the standard; the results were expressed as milligrams of gallic acid equivalents per g sample on a dry weight basis (mg GAE/g dw) through a calibration curve. Measurements were performed in triplicates and averaged.

### 2.4. Spectroscopic Characterization of Flours

#### 2.4.1. Vis–NIR–SWIR Analysis

The Vis–NIR–SWIR measurements of flour samples were performed using a PSR + 3500 spectrometer (Spectral Evolution Inc., Lawrence, MA, USA) operating in the range 350 to 2500 nm. The spectrometer uses one 512-element Si photodiode array for the 350–1000 nm range, with a full width at half maximum (FWHM) resolution of 2.8 nm at 700 nm, a 256-element InGaAs detector covering the 970–1910 nm range with a FWHM resolution of 8 nm at 1500 nm, and finally a second 256-element InGaAs photodiode array for the 1900–2500 nm range having a FWHM resolution of 6 nm at 2100 nm. The measurements were performed at controlled room temperature (25 °C) to ensure thermal stability and consistency across scans. A contact probe was used for all spectral measurements. The sample was manually rotated between scans to increase the scanned surface area and minimize potential spatial heterogeneity. Additionally, prior to scanning, the flour samples were thoroughly mixed to ensure uniform particle size distribution and homogeneity. Five spectral signatures per sample were recorded and averaged to obtain the corresponding reflectance spectral signatures. A Spectralon^®^ diffuse reflectance panel (Labsphere Inc., North Sutton, NH, USA) with 99% reflectance was used to calibrate the spectrometer before measuring each sample.

#### 2.4.2. Spectral Processing Techniques

##### Spectral Pre-Treatments

In addition to using the raw reflectance spectra, we applied a common spectral pre-treatment by calculating the second derivative of reflectance using the Savitzky–Golay filter with the following parameters: a polynomial order of 3 and a window length of 51 nm. Derivative spectroscopy is widely used to enhance spectral resolution and mitigate baseline variations, making subtle spectral features more distinguishable. The second derivative emphasizes inflection points and sharpens overlapping peaks, improving the robustness and interpretability of predictive models. Unlike the first derivative, which primarily highlights the slopes on either side of absorption features and can sometimes incur small shifts in peak position, the second derivative provides a more direct indication of the actual wavelength of absorption. This makes it especially valuable for linking spectral features to specific chemical bonds or constituents. Additionally, derivative pre-treatments help reduce the influence of scattering effects (e.g., due to particle size distribution) and enhance class separation in spectroscopic analyses.

##### Machine Learning Modeling

We employed three widely used multi-output classification algorithms—k-nearest neighbors (k-NN), decision trees (DT), and random forest (RF)—which are inherently multiclass and capable of predicting multiple outputs simultaneously. k-ΝΝ is a simple instance-based algorithm that classifies. a new data point by considering the class labels of its k-nearest neighbors in the training set [34] and captures local spectral patterns. DT is a non-parametric algorithm that builds a tree-like model by recursively splitting the data into subsets based on the most informative features [35] and provides interpretable rule-based decisions. RF is an ensemble learning method that constructs multiple decision trees and combines their outputs to improve predictive accuracy [36]. These models offer an effective balance of simplicity, interpretability, and suitability for high-dimensional spectral data with limited samples.

For each algorithm, hyperparameters were optimized via grid search using 5-fold random cross-validation on the calibration dataset, which consisted of 240 samples (80% of the total data) that were split using random stratified splitting. The remaining 60 samples (20%) were reserved for final testing. Each stratum is defined as a unique combination of flour type and temperature, resulting in 25 strata (5 flour types × 5 baking temperatures). The predefined hyperparameter grids explored during tuning are summarized in Table 1. The F1 macro score (i.e., average of F1 scores for each class) was used as the evaluation metric to select the best hyperparameters; this metric is detailed fully below. After identifying the best hyperparameters, the models were retrained on the full calibration set and then evaluated on the test set.

To assess the performance of the classifiers, the precision, recall, and F1-score evaluation metrics were employed. In this context, each instance can be associated with one or more labels, and the classifier’s goal is to predict all relevant labels for each instance.

In the context of this study, where each output corresponds to a multi-class classification task (e.g., flour type with labels WF, LF, CF, GSF, OSF), true positives, false positives, and false negatives are computed for each class using a one-vs-rest approach.

Precision measures the proportion of correctly predicted positive labels (true positives) out of all instances that are predicted as positive (both true positives and false positives). It focuses on the accuracy of positive predictions.

(1) Precision=True Po
s
i
t
i
v
e
s(
T
r
u
e 
P
o
s
i
t
i
v
e
s+
F
a
l
s
e 
P
o
s
i
t
i
v
e
s)

High precision indicates that when the classifier predicts a label for an instance, it is likely to be correct. It helps to reduce false positives, i.e., instances that are incorrectly classified as positive.

Recall (also known as sensitivity or true positive rate) measures the proportion of correctly predicted positive labels (true positives) out of all instances that actually belong to the positive class (true positives and false negatives). It focuses on the classifier’s ability to find all relevant positive instances.

(2) $$Recall=\frac{\left(True\;Positives\right)}{\left(True\;Positives+False\;Negatives\right)}$$

High recall indicates that the classifier is good at capturing most of the positive instances. It helps to reduce false negatives, i.e., instances that are incorrectly classified as negative when they should be positive.

The F1-score is the harmonic mean of precision and recall and provides a balance between the two metrics. It is a single score that summarizes both precision and recall. The F1-score penalizes models that have imbalanced precision and recall values.

(3)
F
1
s
c
o
r
e=
2×(
P
r
e
c
i
s
i
o
n×
R
e
c
a
l
l)(
P
r
e
c
i
s
i
o
n+
R
e
c
a
l
l)

The F1-score reaches its best value at 1 (perfect precision and recall) and its worst value at 0. It is particularly useful when the dataset is imbalanced, and there is a significant difference between the number of positive and negative instances.

In multi-output multi-class classification, these metrics can be calculated independently for each output, and then micro-averaged or macro-averaged to obtain an overall evaluation score for the entire task. Micro-averaging considers each instance equally, while macro-averaging gives equal weight to each output regardless of its frequency in the dataset.

##### Explainability Analysis

Explainability is a pivotal facet of eXplainable Artificial Intelligence (XAI) that holds significant importance in enhancing trust and transparency in AI systems [37]. In creating AI models that are both powerful and accountable, the most common method employed is ascribing importance scores to the features, i.e., quantifying the influence of each input feature to the model’s inference process. This enables us to dissect and comprehend the underlying decision-making processes of complex AI algorithms by elucidating which features are driving AI model predictions. We empower stakeholders to gain deeper insights into system behavior, facilitate model debugging, and, most importantly, ensure that AI systems are accountable, ethical, and interpretable.

In principle, there are three methodologies to provide feature importance scores [38]. The first approach are a priori methods, employing statistical techniques to assess feature importance independently from developing a machine learning-based model, thereby providing a foundational understanding of feature relevance before model deployment. The other two approaches involve first building a machine-learning based model, and then examining the importance it ascribes to each feature and are thus considered post-hoc. In particular, the second approach, post-hoc model-specific metrics, involves leveraging model-specific information to gauge feature importance and is usually applied in glass-box or opaque models that exhibit inherent interpretability features. Techniques like Variable Importance in Projection (VIP) scores and feature importance derived from model trees capitalize on the unique characteristics of a particular model to quantify feature influence. Lastly, post-hoc model-agnostic metrics, exemplified by the Shapley values [39], offer a model-agnostic perspective on feature attribution, permitting a broader applicability across various AI models including black-box models with limited inherent interpretability.

In this paper, we employed the mutual information criterion as an a priori feature importance methodology to identify the important wavelengths for each output property. The mutual information criterion for feature importance is a measure utilized to assess the significance of individual features in predicting the target variable. It quantifies the information content provided by a feature with respect to the target variable by evaluating the degree of dependency between them. Mutual information captures both the intrinsic relevance of a feature and any potential interactions or dependencies it may exhibit with other features. A higher mutual information score indicates a higher degree of information transfer from the feature to the target variable, thus signifying its greater relevance and predictive value [40].

Moreover, considering that both the decision trees and the random forest model are glass-box models, the feature importance of these models was also calculated using post-hoc techniques. In particular, it is based on the reduction in impurity (often measured by Gini impurity or entropy) achieved by splitting nodes using a particular feature. Features that lead to significant reductions in impurity when used for node splitting are considered more important. In random forests, feature importance is aggregated across multiple trees, reflecting the average decrease in impurity they collectively achieve. Higher feature importance scores indicate that a feature plays a more critical role in decision-making within the model.

##### Spectral Range Selection and Processing Workflow

**Figure 1:**
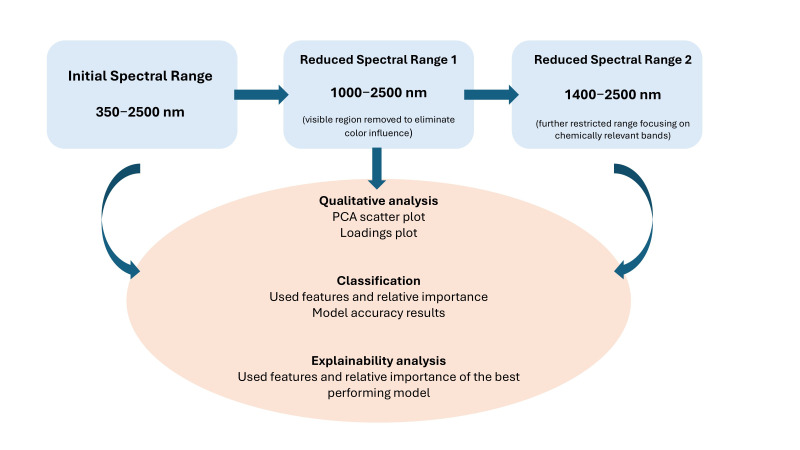
Spectral range refinements and processing workflow.

To identify the most optimal feature subset and develop robust models that properly associate the spectra with the phenolics, we examined different spectral ranges (feature subsets). The goal was to ensure that AI models were trained on infrared regions that reflect meaningful chemically relevant absorption features rather than other characteristics (e.g., visible color). Therefore, spectral refinement was performed to eliminate potential confounding effects or indirect associations, reducing the risk of the models relying on color-related spectral features to infer phenolic content. All spectra were preprocessed using the second derivative, which reduces baseline drift and particle size effects. The spectral range refinement workflow along with the spectral processing methodology are summarized in Figure 1. This design allowed us to assess whether high model accuracy was retained when the visible region (associated with flour color) was excluded while preserving informative overtones and combination bands for water and phenolics. The further reduced spectral range (1400–2500 nm) maintains the SWIR region and excludes residual edge effects while sharpening model specificity toward bands associated with bound water, C–H, and O–H combinations relevant to flour matrix composition. Qualitative analysis involved generating PCA scatter plots and loadings plots to visualize data distribution and identify influential wavelengths within each range. For classification, the models’ performance was evaluated by assessing model accuracy results while the explainability analysis specifically detailed the used features and their relative importance from the best-performing models to understand the driving factors behind the predictions.

### 2.5. Statistical Analysis

All determinations (i.e., chemical and spectral analyses) were performed at least three times for each sample and the mean and standard deviation of the values were calculated. Statistical analyses were carried out using XLSTAT software (version 2021.3.1, Addinsoft, New York, NY, USA). Differences among groups were assessed using two-way analysis of variance (ANOVA). A significance level of α = 0.05 was used for all hypothesis testing procedures, with results considered statistically significant at *p* ≤ 0.05.

Principal Component Analysis (PCA) was applied to the reflectance spectral data to visualize results, explore sample variability and identify clustering patterns. Prior to PCA, spectral data were mean-centered and standardized (autoscaled) to unit variance to eliminate differences in scale and ensure that all variables contributed equally to the analysis. The number of principal components retained (2) was determined based on the cumulative explained variance, aiming to capture at least 95% of the total variance. PCA was performed using the same software environment (XLSTAT), and the results were interpreted through loading plots (Figure S1, Supplementary Material) to evaluate sample distribution and variable contributions.

## 3. Results and Discussion

### 3.1. Color Assesment of Functional Flours

**Figure 2:**
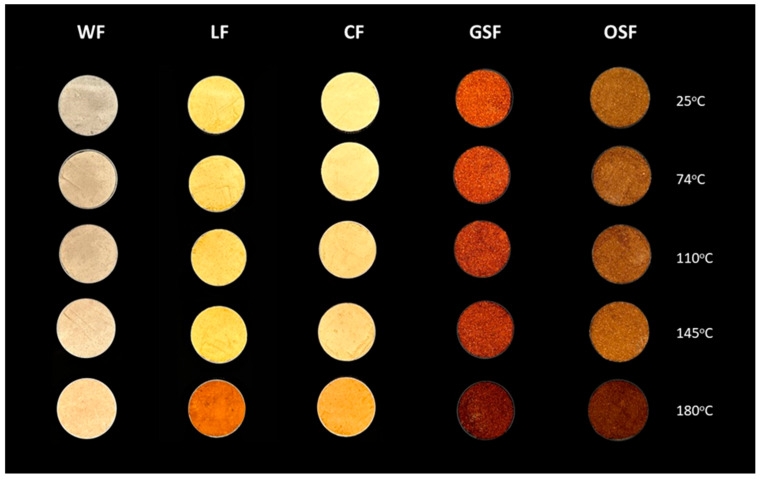
Color differences of flours baked in different temperatures. WF: wheat flour; LF: lupin flour; CF: chickpea flour; GSF; grape seed flour; OSF: olive stone flour.

A progressive darkening of all flour samples was visually observed with increasing temperature (Figure 2), with the most pronounced changes occurring at 180 °C. The color of the flour samples was assessed using the CIELAB color space coordinates (L*, a*, b*) following the thermal treatments at 25, 74, 110, 145, and 180 °C (see Table S2, Supplementary material). The CIELAB analysis revealed statistically significant changes in color parameters across flour types with increasing temperature (Figure S1, Supplementary material). Notably, L* values decreased significantly at ≥145 °C in most flours, indicating darkening due to thermal processing, with lupin, chickpea, and wheat showing the most pronounced reductions (*p* < 0.05). Similarly, a* values significantly increased at 180 °C for chickpea, lupin, and wheat, suggesting the development of reddish-brown tones, while b* values declined significantly at higher temperatures, particularly at 180 °C, reflecting a loss of yellow pigmentation.

### 3.2. Chemical Characterization of Functional Flours

Following color inspection, the total phenolic content (TPC) of the flours was determined. In Table 2, the TPC for each flour with different heat treatment is summarized. GSF at room temperature (25 °C) was found to be rich in phenolic compounds, demonstrating the highest concentration among the tested flours, followed by OSF with significantly high levels of phenolics. Legume flours (LF and CF) had different TPCs, with LF averaging 5.55 mg/g compared to 0.721 mg/g for CF, which comes in agreement with a recent study from Maray (2023) [41]. WF yielded the lowest TPC with an average of 0.662 mg/g. Variations in TPC among flours may be partially attributed to differences in environmental conditions, such as soil composition, climate, and agronomic practices. These factors are known to influence the biosynthesis and accumulation of phenolic compounds in plant tissues and therefore affect the TPC in the derived flours.

Considering the effect of thermal treatment in flours, WF, LF and CF demonstrate a similar concentration-temperature curve, with the phenolic content increasing at 74 °C and then following a decreasing trend up to 110 °C. This initial increase is likely due to the release of bound phenolic compounds as mild heating disrupts cell wall structures and has been previously reported in wheat and oat bran, where TPC increased significantly after thermal treatment at 80 °C [42]. A study by Schefer et al. (2021) [43] on the effects of post-harvest treatment and heat stress (100 °C) on wheat grain flour indicated an increase in phenolics such as ferulic, syringic, vanillic, and p-coumaric acids which could potentially explain the increase of TPC when heated at the lower temperatures. Additionally, processed lupin flour (100 °C for 30 min) has been studied and a significant increase in TPC was found [44]. A different study in chickpea seeds revealed a significant decrease in TPC when heat treatment was applied at various temperatures around 125 °C [45]. While baking temperature increases from 145 °C to 180 °C, a gradual increase in TPC is observed. A study by Rumiyati et al. (2015) [46] on muffins baked at 190 °C with varying substitutions of lupin flour, found to have significantly higher TPC than the unbaked batter which was attributed to the release of phenolic compounds from the cellular structures. Furthermore, earlier research in cereals indicated that a major portion of the total phenolics are present as soluble conjugate or insoluble bound forms [47]. Hence, this increase due to thermal processing could be due to the release of bound phenolic acids from the breakdown of cellular constituents and cell walls in both cereals and legumes. The increase of the TPC in higher temperatures could be also attributed to the separation of conjugate phenolic forms caused by heat treatment, subsequently accompanied by partial polymerization or oxidation of these phenolic components. Another contributing factor might involve phenolics other than those endogenous in the grains, which may be formed as by-products of thermal degradation [48].

Concerning phenolic-rich flours (GSF and OSF), a different trend is observed. TPC is consistent from 25 °C to 110 °C and starts to decrease significantly after 145 °C. The increased temperature required to induce a notable decrease in the overall phenolic content in GSF might be attributed to the elevated concentration of condensed tannins within the seeds. This increased amount of condensed tannins could contribute to a greater resilience against the effects of thermal breakdown. For OSF, a similar resistance against thermal degradation of phenolic compounds is observed which is maintained even at higher temperatures. In contrast, GSF baked at 180 °C illustrated a lower TPC and similar results have been reported by Antony et al. (2022) [49] and Ma et al. (2017) [50] where TPC was drastically reduced after baking at higher temperatures.

From a practical standpoint, evaluating the thermal stability of phenolic compounds is highly relevant for food formulation and processing. This information allows manufacturers to select flours that retain their bioactive properties under heat, optimize baking or drying conditions to minimize nutritional losses, and develop functional food products with improved health value and ingredient performance.

### 3.3. Initial Evaluation Using the Complete Vis–NIR–SWIR Spectral Range (350–2500 nm)

**Figure 3:**
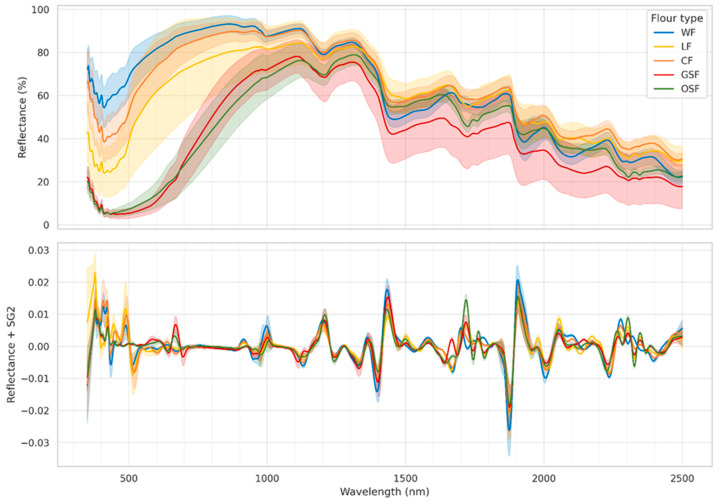
Reflectance spectra for functional flours (**top**) and the second derivative of reflectance per flour type (**bottom**); depicted is the mean spectrum per flour type (solid line) while the shaded part indicates the std.

The mean and standard deviation of the spectral data recorded are depicted in Figure 3 per each flour type; both the initial reflectance data and the second derivative of the reflectance data are visualized. The first derivative of a spectrum is beneficial for removing baseline shifts, which is especially useful in infrared spectroscopy. However, it can create peaks at points where the original spectrum had the steepest slope and zero-crossings where the original had peaks, making interpretation challenging. Infrared spectra often exhibit linear baseline increases, which the second derivative effectively removes. The second derivative of reflectance has negative peaks at the same wavelengths where the original reflectance spectra had peaks, making it more straightforward to understand. For these reasons, second derivatives are frequently favored [51].

The following observations were noted. First of all, WF, CF and LF had the highest mean reflectance in VIS–NIR, while GSF and OSF had very low reflectance in the visible part; this could be attributed to their color [52]. Additionally, there were some common absorption bands across all flour types, such as the 1430 and 1910 nm that are water and –OH related absorption bands, that are commonly found in the literature across various domains including food science [53]. On the other hand, it was also possible to discern some distinct absorption bands for each class. For instance, WF has a noticeable absorbance peak at 2085 nm, which is not found on other flours; similarly, WF differentiates from other flours at 2320 and 2345 nm. OSF and GSF spectra both exhibit sharp absorbance peaks at 1720 nm, which are not found in the other flours. All these indicate that it is possible to classify the flours from Vis–NIR–SWIR spectra.

**Figure 4:**
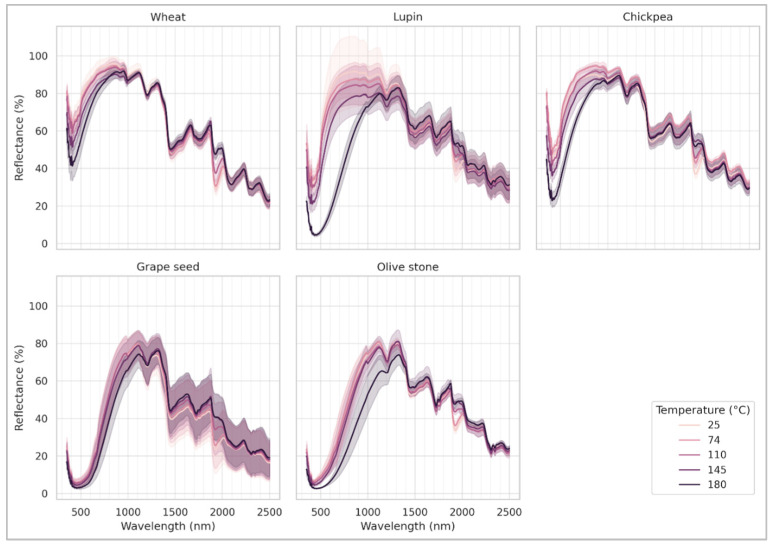
Effect of baking temperature in the reflectance spectra per each flour type.

The effect of baking temperature in the reflectance spectra of each flour type is illustrated in Figure 4. This effect was primarily evident in the visible range for wheat, lupin and chickpea, as expected. There was a noticeable lower albedo (i.e., darker color) as the baking temperature increases. This effect was not apparent in grape seed and olive stone. In these two classes, the most striking effect appears to be the decrease of the absorption bands due to the presence of water as the baking temperature increased; meaning the higher the baking temperature, the more water is lost. The presence of water is most evident at 1920 nm (a combination band of the O–H symmetric stretching band at 3450 cm^−^^1^ and the H–O–H bending at 1640 cm^−1^) and at 1420 nm (2nd overtone of the O–H symmetric stretching band) [54].

**Figure 5:**
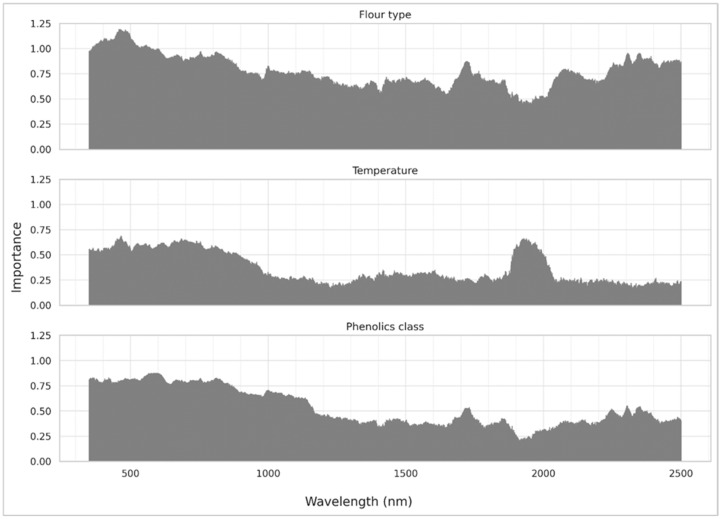
Feature importance using mutual information for each classification dataset using the reflectance spectra.

The a priori relative feature importance scores according to the mutual information criterion per each examined class are presented in Figure 5. A significant portion of the relevant information is contained in the visible range, with evidently the color of the flour presenting a rich source of information pertaining to the flour type and its baked temperature. Another interesting observation is that the baking temperature may be determined (as expected) by the water absorption bands and particularly at 1920 nm, underlying probably the fact that the loss of water is associated with higher baking temperatures. Moreover, to differentiate the flour types, the SWIR region contributes mostly with a series of peaks, indicating the presence of absorption bands that arise from combination bands or overtones of the fundamental frequencies corresponding to the molecular vibrations taking place in the mid-Infrared region. In particular, it should be noted that two distinct broad peaks centered around 2305 and 2345 nm are identified, which can be used to differentiate both the flour type and the concentration of phenolics.

Finally, an important observation is that the distribution of the feature importances of the phenolics class mirrors the one of the flour type, indicating that they may be indirectly determined through cross-correlation, rather than only denoting the actual absorption bands of the phenolic substances, particularly considering that the relative importance ascribed is higher in the visible region than the SWIR region.

### 3.4. Spectral Interpretation and Performance

#### 3.4.1. Qualitative Analysis

**Figure 6:**
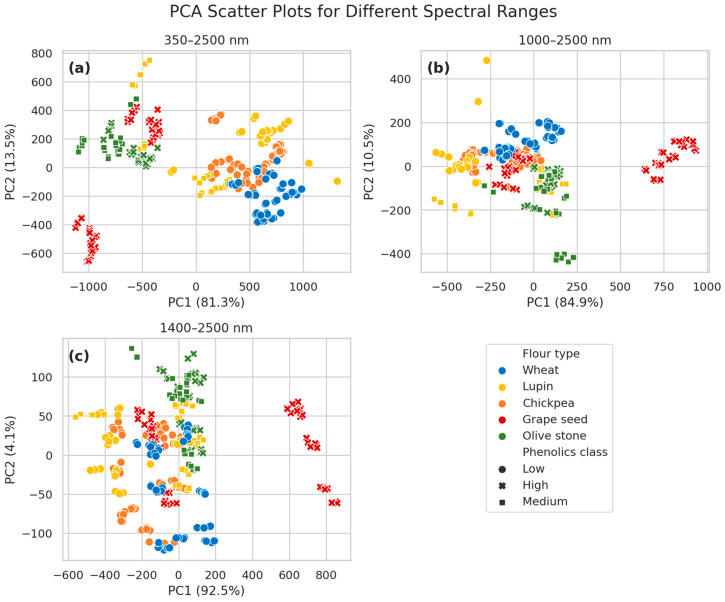
PCA scatter plots of the reflectance spectra corresponding to flours with different thermal treatment, categorized per flour type (color) and phenolics class (shape). Results are shown for three spectral ranges: (**a**) 450–2500 nm, (**b**) 1000–2500 nm, and (**c**) 1400–2500 nm.

In order to explore flour sample similarities, a PCA scatter plot (Figure 6) of the first two principal components per each spectral range examined was created, as developed from the raw reflectance spectra of all the available points. When the complete spectrum is employed (Figure 6a), the first two components explain 94.8% of the total variance and most flour types are easily distinguishable and bundled together into separable clusters (e.g., grape seed, olive stone, and wheat). Thus, the classification task of recognizing the flour type from the reflectance spectra seems to be possible. Moreover, after observing the data distribution, a classification of the TPC was performed and three separate classes were defined: low (0–10 mg/g), medium (10–20 mg/g and high (20–100 mg/g). WF, LF and CF are clustered together due to a low TPC, while GSF and OSF correspond to a higher phenolics class. This supports the hypothesis that phenolic content correlates with flour type and is partially reflected in the spectral variance captured by the PCA.

As the spectral range becomes narrower (Figure 6b,c), the data points become more clustered together. In the 1000–2500 nm spectral region, PC1 and PC2 together still explain 95.4% of the variance (PC1: 84.9%, PC2: 10.5%), and grape seed flour remains highly distinguishable, but the separation between other flour types becomes less distinct. This suggests that removal of color-related spectral features results in some loss of separability, particularly for flour types with similar chemical and physical properties.

When the spectral region is further restricted to the 1400–2500 nm range (Figure 6c), PC1 captures 92.5% of the variance, but PC2 explains only 4.1%, indicating reduced information in this window. Here, most flour types appear increasingly overlapped along PC2, though grape seed flour again stands out as a distinct group. The separation among phenolics classes also becomes less visually apparent. This suggests that while the SWIR region contains sufficient chemical information for classification, the exclusion of visible and NIR wavelengths limits the dimensionality of variance available for unsupervised discrimination.

#### 3.4.2. Classification of Functional Flours from Vis–NIR–SWIR Spectra

Model evaluation was performed based on accuracy results in the independent test set and the results for the best fitting model in each spectral range are summarized in Table 3. Considering the overall classification accuracy, RF yielded the highest score across all three output classes although the accuracy of both kNN and DT algorithms was high, with only a few misclassifications. Interestingly, when examining the precision and recall metrics for each of the classes, it is evident that there is no single class that is systematically misclassified, indicating that the classifiers are robust and showcase a well-rounded performance. The full spectral range (350–2500 nm) produced the highest accuracies, with values near or above 0.98 across flour type, thermal treatment, and phenolics classification. When the visible region was excluded (1000–2500 nm and 1400–2500 nm), flour type and phenolic content classification remained strong (up to 0.98), while thermal treatment classification showed a slight drop in accuracy. Nonetheless, it ought to be noted that the more elaborate RF model attained (marginally higher) accuracy than the kNN approach. Detailed model accuracy results for each spectral range are provided in the Supplementary Material (Tables S3–S5).

To ascertain why the models exhibit high accuracy, it is necessary to examine the results of the interpretability analysis, as shown in the following section. However, to put them into perspective, when compared to other studies, the accuracy levels are similar. For example, in the study of Zhang et al. (2023) [55], the best model to classify five types of wheat flour using the Vis–NIR–SWIR spectra attained an accuracy of 100%. Similarly, in [56], the best model was able to differentiate between pure and adulterated purple sweet potato powder with white potato powder with an accuracy of 100%, while another study that focused on classifying rice flour into two varieties (Indica and Japonica) from NIR spectra attained an accuracy of 91% [57]. With respect to classifying heat treatments, Badaro et al. (2022) [58] and Verdú et al. (2017) [59] demonstrated that there exists a pattern evolution of the flours treated by different heat treatments in wheat and oat flours, respectively, when examining the Vis–NIR spectra (400 to 1000 nm). Finally, using the 1000 to 2500 nm range, Tian et al. (2021) [24] developed a regression model to predict the phenolics in wheat flour with an excellent fit corresponding to an R^2^ of 0.90.

#### 3.4.3. Explainability Analysis from Vis–NIR–SWIR Spectra

**Figure 7:**
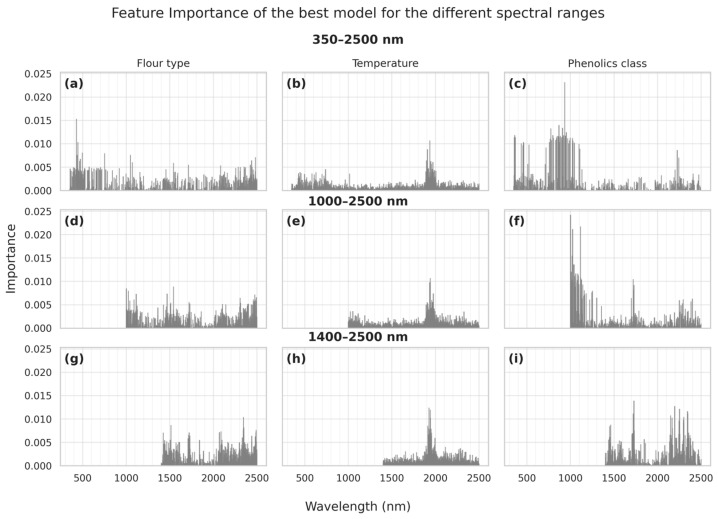
Used features and their relative importance according to the best model for each classification dataset and spectral range. Top row (sublots (**a**–**c**)) illustrates feature importance when utilizing the 350–2500 nm spectral range for classifying (**a**) Flour type, (**b**) Temperature, and (**c**) Phenolics class. Middle row (subplots (**d**–**f**)) shows the corresponding feature importance when the 1000–2500 nm range is employed for (**d**) Flour type, (**e**) Temperature, and (**f**) Phenolics class. The bottom row (subplots (**g**–**i**)) details feature importance for (**g**) Flour type, (**h**) Temperature, and (**i**) Phenolics class, derived from the more restricted 1400–2500 nm spectral range.

The results of the post-hoc interpretability analysis of the best models are depicted in Figure 7, which denotes the feature importances as identified by the best model per each of the three output classes. Interestingly, the general distribution denotes some sharp peaks and it is possible to identify some key absorption bands with the model efficiently performing feature selection.

The flour type, when the complete spectral range is used (Figure 7a), is mostly determined from its color (sharp peaks between 350 and 500 nm), while two additional areas in which the model focuses on are the 1450 nm (adjacent to a water and –OH absorption band) and the 2300 and 2400 nm range. As also noted in the qualitative analysis, this region is rich with bands that differentiate the flour types. As we limit the spectral range and force the models to concentrate in the NIR (Figure 7d) and SWIR (Figure 7g), some notable features noted are at around 2320 nm, that may be potentially attributed to the presence of starch and particularly the vibrational modes of amylose and amylopectin, its main components, which give rise to a fundamental absorption band at 1077 cm^−^^1^ [60], and thus can be used to identify WF. This contrasts with the absorptions noted for the other flours that are found around 2305 nm, corresponding probably to the third overtone of the aromatic C–C stretching bond at ca. 1443 cm^−1^ [61,62] and a combination band of the second overtone of the stretching of C=OO− and aromatic C=C groups at 1600 cm^−1^, and the aromatic C–H stretching at 1143 cm^−1^, both related to phenolic compounds [63]. Similarly, the absorption at 2345 nm which differentiates WF from other flours could be attributed to combinations from the fingerprint region of phenol, and particularly of the sharp absorption at 1500 cm^−1^ and the absorption at 1380 cm^−1^ attributed to C–O stretching vibrations [64]. Less important absorption bands identified by the model may be noted around 2100 nm. These may be attributed to the fundamental absorption band at 4800 cm^−1^ of the C–O–O bond, and the wheat flour proteins like amide I and amide II that have distinct absorption bands at 1650 cm^−1^ and 1540 cm^−1^ (CN stretching coupled to NH bending), whose combination gives rise to an absorption band at about 2100 nm [65,66]. A less sharp peak at 2140 nm may be attributed to the combination of the asymmetric stretching vibration of the CH_2_ groups and the fundamental at 1744 cm^−1^ arising from the absorption of the C=O bonds of the ester groups, which is due to the presence of the fatty acids and their glycerides [67]. Some final identified bands are around 1700 nm; the absorbance at 1660 nm may be due to a combination band at 6016 cm^−1^, which arises from the third overtone of a sharp peak at the fingerprint region at 1031 cm^−1^ from the C–O stretching bond and an absorption at 2923 cm^−1^ from the asymmetric stretching vibrations of CH_2_ groups [61]. On the other hand, the 1720 nm absorbance may be due to both a combination band from fundamental absorptions of the asymmetric and symmetric stretching vibrations of CH_2_ groups (at 2923 cm^−1^ and 2853 cm^−1^, respectively) and their respective second overtones [68].

With respect to the temperature, as expected, it is mostly determined from the flour’s color and (most importantly) the sharp broad water absorption band at 1920 nm, which dominates across all spectral ranges (Figure 7b,e,h). This is consistent with all previous observations, but it is important to note that the broad water absorption band has higher relative importance which may contribute to a more robust model.

Finally, the phenolics class in the case when the complete spectral range is used (Figure 7c) is identified mostly by the visible part and near infrared, with very high importance from a broad region centered around 900 nm, and with less importance by the bands around 1020 nm and the 2150 to 2300 nm in SWIR. With respect to the visible part, it should be noted that the different flour types which can be classified by their color, have also different phenolic concentrations (i.e., WF has fewer phenolics). Thus, these bands may be used to identify the phenolics class with this inter-correlation. The area around 900 nm may be due to the third overtone of the fundamental 3800 cm^−1^ broad absorbance of phenol–OH [69] and has been reported in FTIR spectra of various oil seeds [24]. As we limit the spectral range and force the models to concentrate in the NIR (Figure 7f) and SWIR (Figure 7i), we can identify some absorbances in the SWIR, which may be tied to phenolic compounds [70]. For example, the absorption peaks between 2100 and 2300 nm could be ascribed to the 3rd overtones of the strong absorption bands at 1480, 1500, and 1600 cm^−1^ which are the usual aromatic bands (C=C arene). In phenol itself, monosubstituted aromatic-ring peaks are visible at the fingerprint region at 690 and 760 cm^−1^ which when combined with the 3500 cm^−1^ of the –OH group may be noted at 2.3 to 2.4 μm. Finally, the quite important band identified at 1.72 μm may be due to a combination of the 2nd overtones at 1500 and 1600 cm^−1^ of the aromatic bands and the 2nd overtone of the O–H in-plane bending vibration centered at 1380 cm^−1^. Nevertheless, it must be noted that given the very high importance ascribed to one spectral region (i.e., around 900 nm) and the flours’ color, the robustness of this model may be questioned, particularly if it is applied in different flour types not seen by the model which may have overlapping absorptions near the 900 nm and/or different color.

### 3.5. Study Strengths, Limitations and Future Directions

This study demonstrates the potential of integrating Vis–NIR–SWIR spectroscopy with machine learning for classifying functional flours based on thermal treatment, flour type, and phenolic content. A key strength of the work lies in the stepwise spectral reduction approach where, progressively, spectral bands were excluded to evaluate their contribution to classification performance. Additionally, the non-destructive and rapid nature of this spectroscopic technique offers a valuable advantage over traditional analytical methods, particularly for in-line quality control in the food industry. Nevertheless, there are several limitations that should be considered. The classification accuracy for phenolic content is apparently influenced in part by indirect correlations such as flour color and type, that may weaken models if they are to be applied to diverse types of flour. Furthermore, the dataset includes a limited range of flour types and thermal treatments, which may constrain the generality of the findings. Although efforts were made to reduce the influence of color—through spectral refinement and second derivative preprocessing—the dataset still lacks samples that would allow a complete decoupling of phenolic content from visual appearance (e.g., dark-colored flours with low TPC or light-colored flours with high TPC). This limitation hinders the ability to fully isolate chemically relevant spectral features from those indirectly associated with color. Additionally, the results of the Folin–Ciocalteu assay should be interpreted with caution, as the method is non-specific and responds to a broad range of reducing substances beyond phenolics. Future research would benefit from incorporating more selective analytical techniques, such as LC-MS/MS, to accurately quantify individual phenolic compounds. In parallel, future studies should also prioritize the expansion of the flour spectral library to a broader variety of matrices and admixtures along with processing conditions to increase model robustness. Finally, targeted feature selection techniques could be employed to isolate specific, chemically relevant spectral bands, which can then be used to retrain models for greater precision and reduced reliance on matrix-dependent characteristics.

## 4. Conclusions

Functional flours are rich in phenolic compounds and could be used to produce novel and nutritious foods. The thermal treatment of flours delivered promising results in terms of maintaining their respective phenolic compound concentration during processing. Legume flours and WF illustrated an increase in TPC when mild heating (74 °C) was applied, which could be beneficial for producing added-value products to consumers. GSF and OSF were observed to contain more thermally stable compounds, even at high baking temperatures, which would be ideal for incorporating into recipes requiring more intense thermal treatment. In the current study, Vis–NIR–SWIR spectroscopic techniques were deployed, attempting to classify the available samples by flour category, thermal treatment, and phenolic compound concentration. A stepwise spectral range reduction approach was followed to assess the accuracy of the classification models where the full 350–2500 nm range was initially examined and then spectral regions were progressively removed; first eliminating the visible region (1000–2500 nm), then narrowing further to the SWIR-only region (1400–2500 nm). The Random Forest model delivered strong classification performance for the global classification of flour type, heat treatment and phenolic content across all spectral windows. The multi-output classification model was able to identify certain wavelengths along with their relative importance and indicate where key features lie for each classification dataset, assisting relevant studies to connect chemometrics to spectral data.

The explainability analysis provided insight into the wavelengths used by the RF model and justified the high accuracy for detecting the flour type and the baked temperature by noting bands which may be tied to overtones and combinations of fundamental absorptions in the NIR. While high accuracy was observed for phenolic content classification, the analysis suggests that this performance may be influenced in part by indirect factors—such as flour type and color—which are correlated with phenolic concentration. Although color-related effects were reduced through spectral refinement and derivative preprocessing, the dataset lacks flours with fully decoupled visual appearance and phenolic levels, limiting the ability to isolate chemical contributions with full certainty.

The results of this paper support the perspective for the potential use of rapid and non-destructive spectroscopic techniques to enable quality control and quick classification of flours by the food industry. The findings of this study may be also extended to other flour-like ingredients derived from agricultural by-products, highlighting the potential of such materials as functional food components rich in phenolic compounds. Given the different nature of the available functional flours and their respective chemical composition, the creation of reference databases/libraries, along with more phenolic-specific validation methods, are the most essential conditions for building robust classification models able to assist time and labor-consuming analyses by the food industry. Further data collection and additional studies are necessary to ascertain whether phenolic content can be reliably identified and accurately quantified using Vis–NIR–SWIR spectroscopy.

## Figures and Tables

**Table 1:** The grid search space used for tuning the hyperparameters of each learning algorithm. *M* is the total number of available features.

Model	Hyperparameter	Values Tested
k-NN	Number of neighbors (k)	[1,20]
	Distance metric	Euclidean and Cosine
Decision Tree	Max features	{ M, l o g 2( M), M}
Random Forest	Max features	{ M, l o g 2( M), M}
	Number of estimators	{10, 50, 100, 200}

**Table 2:** Mean Total Phenolic Content (TPC) of flours with different heat treatment expressed in mgGAE/g flour.

°C (Mean ± SD)	WF	CF	LF	GSF	OSF
25 °C	0.662 ± 0.065 ^a^	0.721 ± 0.005 ^a^	5.547 ± 0.348 ^a^	88.121 ± 2.393 ^c^	18.774 ± 1.177 ^a^
74 °C	0.852 ± 0.072 ^a^	0.787 ± 0.004 ^a^	7.320 ± 0.699 ^a^	91.807 ± 3.436 ^c^	19.885 ± 1.011 ^a^
110 °C	0.541 ± 0.145 ^a^	0.528 ± 0.027 ^a^	5.276 ± 0.004 ^a^	91.590 ± 0.948 ^c^	18.684 ± 1.382 ^a^
145 °C	0.543 ± 0.038 ^a^	0.565 ± 0.039 ^a^	4.562 ± 0.012 ^a^	79.677 ± 0.355 ^c^	17.886 ± 0.742 ^a^
180 °C	0.745 ± 0.023 ^a^	0.669 ± 0.014 ^a^	5.621 ± 0.515 ^a^	60.912 ± 2.630 ^b^	15.713 ± 1.036 ^a^

WF: wheat flour; LF: lupin flour; CF: chickpea flour; GSF; grape seed flour; OSF: olive stone flour; Mean values followed by different superscript letters within the table are significantly different from each other based on Tukey’s HSD post-hoc test (*p* < 0.05).

**Table 3:** Accuracy results of the best performing multi-output classification algorithm (kNN, DT, RF) for the three sets of output classes (flour type, temperature, phenolics) in the tested spectral range.

Class	350 to 2500 nm			1000 to 2500 nm			1400 to 2500 nm		
	Prec.	Recall	F1-Score	Prec.	Recall	F1-Score	Prec.	Recall	F1-Score
**Flour type**									
Wheat	1.00	0.99	0.99	1.00	0.82	0.90	0.91	0.91	0.91
Lupin	0.98	0.98	0.98	0.97	0.98	0.98	0.98	0.98	0.98
Chickpea	0.97	0.98	0.98	0.97	0.97	0.97	0.92	0.92	0.92
Grape seed	0.98	0.98	0.98	0.98	0.96	0.97	0.98	0.98	0.98
Olive stone	0.98	0.98	0.98	0.96	0.98	0.97	0.98	0.98	0.98
**accuracy**			**0.98**			**0.96**			**0.95**
**Temperature**									
25	0.98	0.98	0.98	0.92	0.92	0.92	1.00	0.92	0.96
74	0.98	0.98	0.98	0.85	0.85	0.85	0.85	0.85	0.85
110	0.98	0.97	0.97	0.79	0.85	0.81	0.77	0.77	0.77
145	0.97	0.98	0.98	0.60	0.75	0.67	0.58	0.88	0.70
180	0.98	0.98	0.98	0.90	0.69	0.78	0.80	0.62	0.70
**accuracy**			**0.98**			**0.82**			**0.80**
**Phenolics**									
Low	0.99	0.99	0.99	0.99	0.99	0.99	0.99	0.99	0.99
Medium	0.97	0.98	0.98	0.94	0.97	0.95	0.94	0.98	0.95
High	1.00	0.99	0.99	0.99	0.98	0.98	0.99	0.98	0.98
**accuracy**			**0.99**			**0.98**			**0.98**
**Best model and optimal hyperparameters**									
	**Random Forest with Ref.** **Max. feat.=** $\sqrt{M}$, est. = 100			**Random Forest with Ref. + SG2** **Max. feat.=** ${log}_{2}(M)$, est. = 50			**Random Forest with Ref. + SG2** **Max. feat.=** $\sqrt{M}$, est. = 100		

## Data Availability

The data that supports the findings of this study are available from the corresponding author upon reasonable request.

## Supplementary Materials

The following supporting information can be downloaded at: https://www.mdpi.com/article/10.3390/foods14152663/s1, Figure S1: Boxplots illustrating the effect of temperature on CIELAB color parameters (L*, a*, b*) across different flour types. Letters above boxes denote statistical significance (ANOVA with Tukey HSD, α=0.05); groups sharing a letter are not significantly different; Figure S2: Loadings plot for WF, LF, CF, GSF and OSF flours in different thermal treatments from 350–2500 nm corresponding to PCA; Figure S3: PCA loadings from the 1000–2500 nm spectral range for WF, LF, CF, GSF and OSF flours in different thermal treatments; Figure S4: PCA loadings from the 1400–2500 nm spectral range for WF, LF, CF, GSF and OSF flours in different thermal treatments; Table S1: Granulometric composition of wheat, lupin, chickpea, grape seed and olive stone flours; Table S2: CIELAB coordinates of flours in different thermal treatments; Table S3: Accuracy results in the independent set using three multi-output classification algorithm (kNN, DT, RF) for the three sets of output classes (flour type, temperature, phenolics) in the 350–2500 nm spectral range; Table S4: Accuracy results in the independent set using three multi-output classification algorithm (kNN, DT, RF) for the three sets of output classes (flour type, temperature, phenolics) in the 1000–2500 nm spectral range; Table S5: Accuracy results in the independent set using three multi-output classification algorithm (kNN, DT, RF) for the three sets of output classes (flour type, temperature, phenolics) in the 1400–2500 nm spectral range.
