# Supplementary material for: Evaluating the Effect of Thermal Treatment on Phenolic Compounds in Functional Flours Using Vis–NIR–SWIR Spectroscopy: A Machine Learning Approach
Source: Foods. 2025 Jul 29;14(15):2663. doi: 10.3390/foods14152663 (PMC12346101; doi:10.3390/foods14152663)
Supplement: Supplementary file 1 [file foods-14-02663-s001.zip › foods-3630535-supplementary.pdf]

Supplementary material

Assessing the Influence of Thermal Treatment on Phenolic Compounds Content in Functional Flours through Visible, Near- and Shortwave-Infrared (VNIR–SWIR) Spectroscopy

Table S1. Granulometric composition of wheat, lupin, chickpea, grape seed and olive stone flours.

Mean particle size (μm)	WF (%)	LF (%)	CF (%)	GSF (%)	OSF (%)
**9**	6.49	0.37	0.35	0.30	0.23
**79.5**	18.66	4.86	1.20	1.98	0.36
**175**	4.43	2.16	1.87	1.52	0.68
**250**	21.08	6.79	2.38	6.75	2.18
**350**	1.14	10.85	4.46	2.21	10.55
**500**	0.68	36.72	16.24	20.73	75.03
**600**	47.52	38.26	73.50	66.50	10.98

*WFC: wheat flour commercial; WFL: wheat flour Lemnos; LFC: lupin flour commercial; LFL: lupin flour Lemnos; CFC: chickpea flour commercial; CFL: chickpea flour Lemnos; GSFC: grape seed flour commercial; GSFL: grape seed flour Lemnos; OSFC: olive stone flour commercial; OSFL: olive stone flour Lemnos

Table S2. CIELAB coordinates of flours in different thermal treatments

Flour type	Temperature	L*_mean	L*_std	a*_mean	a*_std	b*_mean	b*_std
**CF**	25	2225.763301	14.669957	20.585529	7.193693	446.816864	45.960820
**CF**	74	2274.840661	12.861384	10.831562	7.887038	439.384438	45.749803
**CF**	110	2207.072666	59.279227	25.245035	11.457494	472.338794	20.305689
**CF**	145	2129.618087	55.567114	41.995723	16.628968	483.807873	19.103381
**CF**	180	1909.374020	61.855585	133.593159	36.006663	551.978119	44.622141
**GSF**	25	1116.311648	152.068295	309.128067	27.513078	295.646407	37.812449
**GSF**	74	1121.373160	140.119987	302.962839	32.236461	273.829771	46.909005
**GSF**	110	1101.304445	131.302377	300.510434	27.248115	288.360529	32.969126
**GSF**	145	1048.740626	136.938693	300.973940	33.199118	295.529608	65.452751
**GSF**	180	901.292970	105.738816	270.685843	54.671006	218.805031	95.291741
**LF**	25	2210.243102	115.969160	-2.685803	24.046857	735.719214	181.769355
**LF**	74	2187.698552	65.398502	-6.899631	17.791053	658.900401	9.152956
**LF**	110	2142.892002	78.475445	6.949969	14.437198	679.802228	16.500156
**LF**	145	1986.472384	104.408966	87.685958	10.117861	699.022721	40.703780
**LF**	180	1206.984868	88.967791	310.151871	21.174808	528.109518	59.177115
**OSF**	25	1273.614031	133.501493	189.506257	59.809297	394.279356	138.779965
**OSF**	74	1272.196136	126.519073	191.823951	61.632763	398.515787	128.858012
**OSF**	110	1217.289305	102.668979	197.618435	68.579444	397.368633	122.295219
**OSF**	145	1144.632696	63.013358	221.666284	85.603706	408.650580	127.601942
**OSF**	180	890.911690	45.475823	214.187392	71.868102	245.169514	64.587909
**WF**	25	2335.027922	46.791476	-0.412919	9.990296	265.807210	42.721123
**WF**	74	2314.738837	36.426652	1.057556	21.710085	253.174974	51.529197
**WF**	110	2306.552629	34.293672	5.324030	4.417496	254.852875	43.213622
**WF**	145	2239.370143	35.961672	19.456029	7.573222	281.766376	37.637808
**WF**	180	2147.489212	73.579872	61.507653	16.270991	371.921626	66.853290

***WF: Wheat flour, LF: Lupin flour, CF: Chickpea flour, GSF: Grape seed flour, OSF: Olive stone flour**

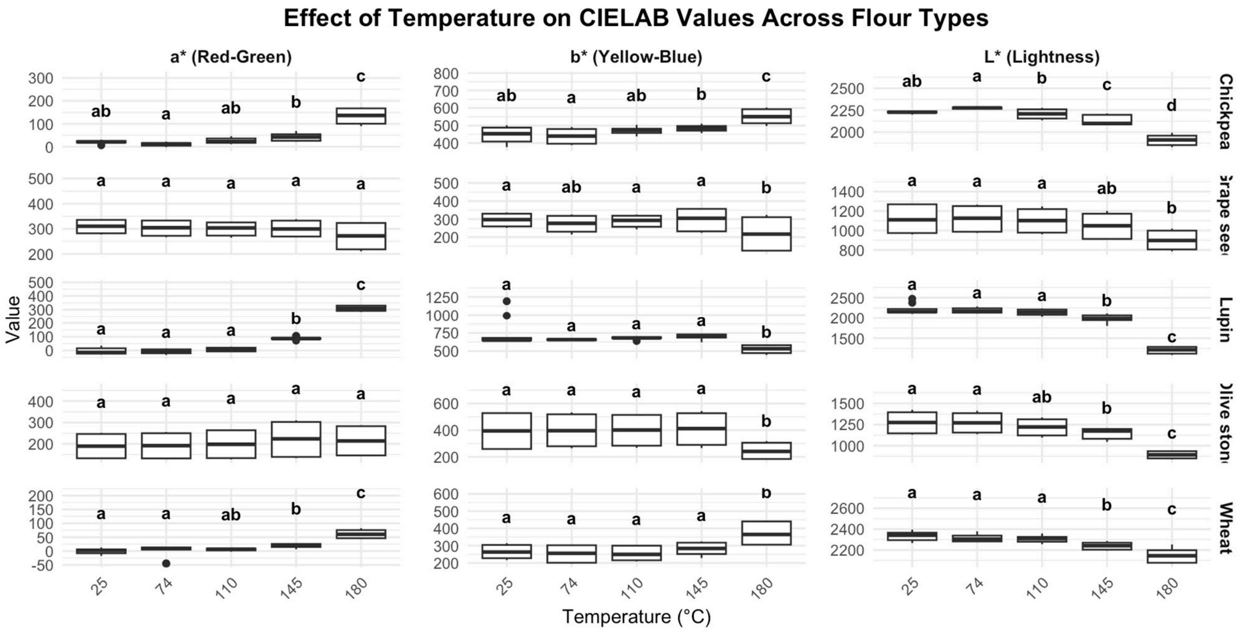

Figure S1. Boxplots illustrating the effect of temperature on CIELAB color parameters (L*, a*, b*) across different flour types. Letters above boxes denote statistical significance (ANOVA with Tukey HSD, α=0.05); groups sharing a letter are not significantly different.

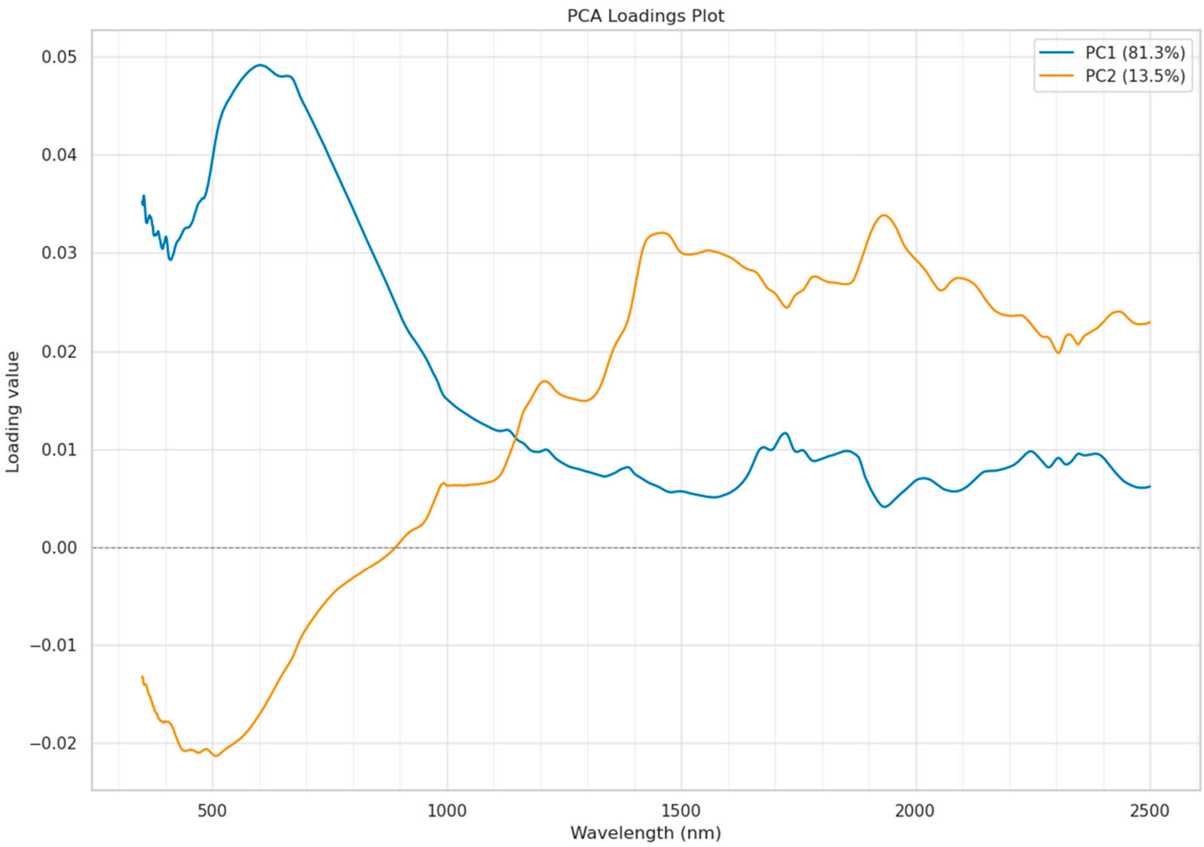

Figure S2. Loadings plot for WF, LF, CF, GSF and OSF flours in different thermal treatments from 350-2500 nm corresponding to PCA

***WF: Wheat flour, LF: Lupin flour, CF: Chickpea flour, GSF: Grape seed flour, OSF: Olive stone flour**

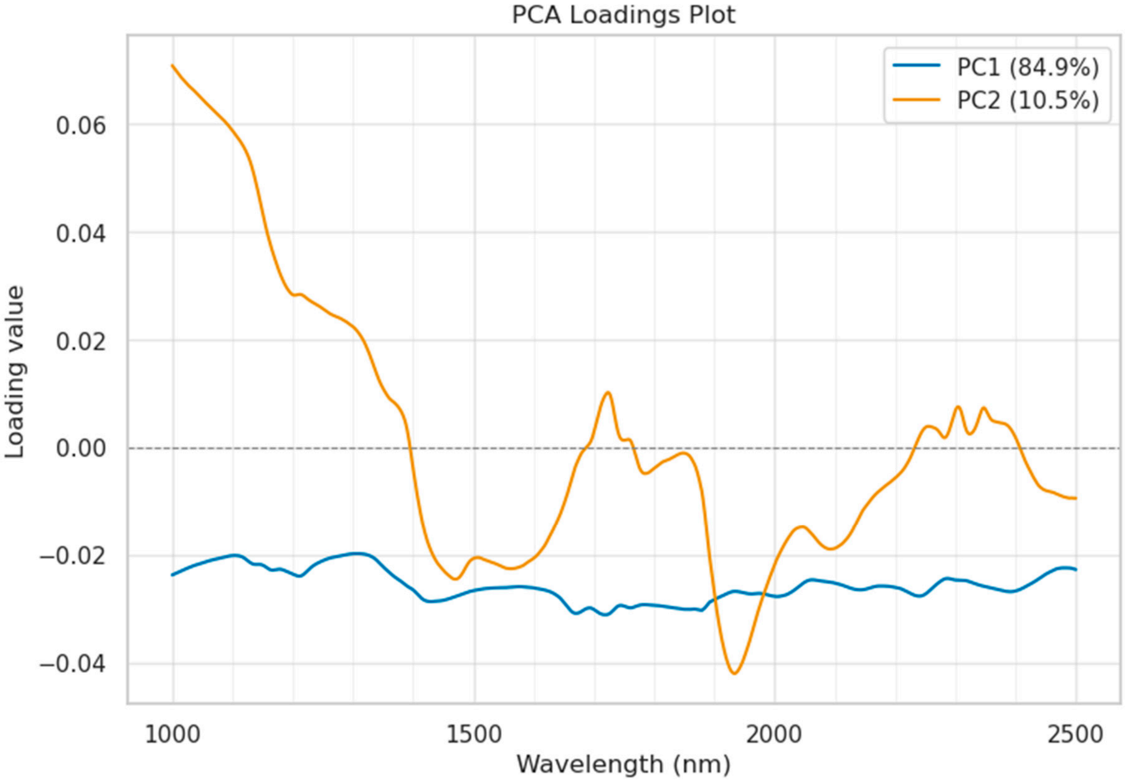

Figure S3. PCA loadings from the 1000-2500 nm spectral range for WF, LF, CF, GSF and OSF flours in different thermal treatments.

***WF: Wheat flour, LF: Lupin flour, CF: Chickpea flour, GSF: Grape seed flour, OSF: Olive stone flour**

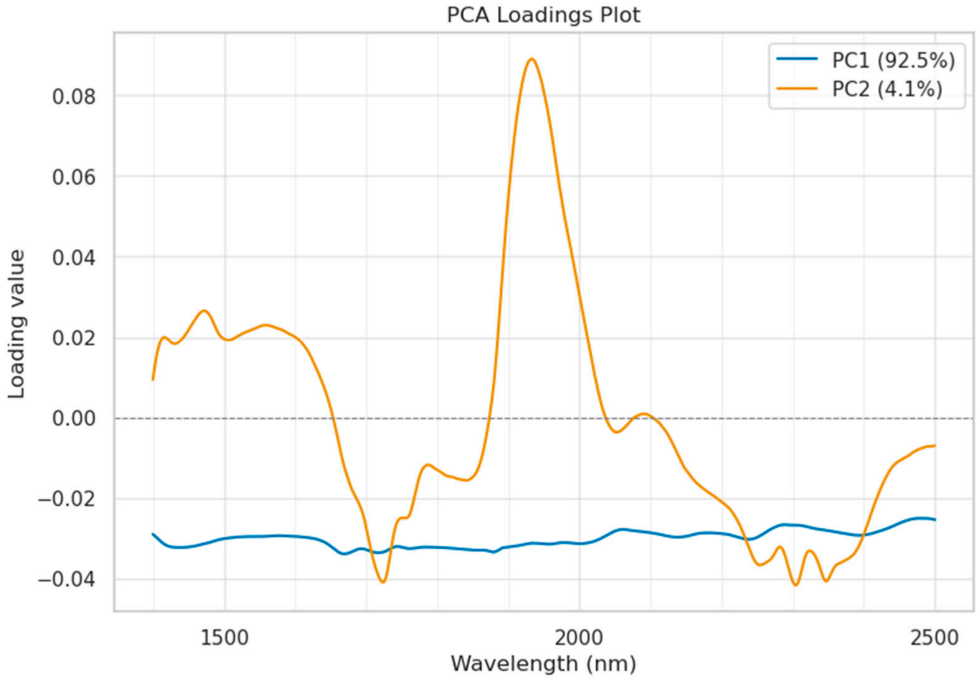

Figure S4. PCA loadings from the 1400-2500 nm spectral range for WF, LF, CF, GSF and OSF flours in different thermal treatments.

***WF: Wheat flour, LF: Lupin flour, CF: Chickpea flour, GSF: Grape seed flour, OSF: Olive stone flour**

Table S3. Accuracy results in the independent set using three multi-output classification algorithm (kNN, DT, RF) for the three sets of output classes (flour type, temperature, phenolics) in the 350–2500 nm spectral range.

	**kNN**			**DT**			**RF**		
Class	Prec.	Recall	F-score	Prec.	Recall	F-score	Prec.	Recall	F-score
**Flour type**									
Wheat	1.00	0.97	0.99	1.00	0.99	0.99	1.00	0.99	0.99
Lupin	0.97	0.98	0.98	0.97	0.98	0.98	0.98	0.98	0.98
Chickpea	0.95	0.97	0.96	0.97	0.97	0.97	0.97	0.98	0.98
Grape seed	0.96	0.96	0.96	0.98	0.96	0.97	0.98	0.98	0.98
Olive stone	0.96	0.96	0.96	0.96	0.98	0.97	0.98	0.98	0.98
**accuracy**			**0.97**			**0.98**			**0.98**
**Temperature**									
25	0.96	0.96	0.96	0.96	0.96	0.96	0.98	0.98	0.98

74	0.97	0.97	0.97	0.98	0.97	0.97	0.98	0.98	0.98
110	0.95	0.97	0.96	0.97	0.97	0.97	0.98	0.97	0.97
145	0.95	0.95	0.95	0.97	0.98	0.98	0.97	0.98	0.98
180	0.98	0.97	0.98	0.98	0.98	0.98	0.98	0.98	0.98
**accuracy**			**0.96**			**0.97**			**0.98**
**Phenolics**									
Low	0.99	0.99	0.99	0.99	0.99	0.99	0.99	0.99	0.99
Medium	0.95	0.97	0.96	0.94	0.97	0.95	0.97	0.98	0.98
High	0.99	0.98	0.98	0.99	0.98	0.98	1.00	0.99	0.99
**accuracy**			**0.98**			**0.98**			**0.99**
**Best model and optimal hyperparameters**									
	**Ref.**<br>**k=9, dist.=Euclidean**			**Ref.**<br>**Max feat.=** $\sqrt{M}$			**Ref.+SG2**<br>**Max. feat.=** $log_2(M)$, est.=50		

Table S4. Accuracy results in the independent set using three multi-output classification algorithm (kNN, DT, RF) for the three sets of output classes (flour type, temperature, phenolics) in the 1000–2500 nm spectral range.

	kNN			DT			RF		
Class	Prec.	Recall	F-score	Prec.	Recall	F-score	Prec.	Recall	F-score
**Flour type**									
Wheat	1.00	1.00	1.00	0.82	0.82	0.82	1.00	0.82	0.90
Lupin	0.90	1.00	0.95	0.82	1.00	0.90	0.97	0.98	0.98
Chickpea	1.00	1.00	1.00	0.75	0.75	0.75	0.97	0.97	0.97
Grape seed	1.00	1.00	1.00	1.00	0.89	0.94	0.98	0.96	0.97
Olive stone	1.00	0.90	0.95	1.00	1.00	1.00	0.96	0.98	0.97
**accuracy**			**0.98**			**0.88**			**0.96**
**Temperature**									
25	0.73	0.85	0.79	0.73	0.85	0.79	0.92	0.92	0.92

74	0.71	0.77	0.74	0.71	0.77	0.74	0.85	0.85	0.85
110	1.00	0.92	0.96	1.00	0.69	0.82	0.79	0.85	0.81
145	0.62	1.00	0.76	0.73	1.00	0.84	0.60	0.75	0.67
180	0.91	0.77	0.83	0.91	0.77	0.83	0.90	0.69	0.78
**accuracy**			**0.80**			**0.80**			**0.82**
**Phenolics**									
Low	0.99	0.99	0.99	0.99	0.99	0.99	0.99	0.99	0.99
Medium	0.92	0.97	0.95	0.94	0.97	0.95	0.97	0.98	0.98
High	0.99	0.96	0.98	0.99	0.98	0.98	1.00	0.99	0.99
**accuracy**			**0.98**			**0.98**			**0.99**
**Best model and optimal hyperparameters**									
	**Ref.**<br>**k=9, dist.=Euclidean**			**Ref.**<br>**Max feat.=** $\sqrt{M}$			**Ref.+SG2**<br>**Max. feat.=** $\sqrt{M}$, est.=100		

Table S5. Accuracy results in the independent set using three multi-output classification algorithm (kNN, DT, RF) for the three sets of output classes (flour type, temperature, phenolics) in the 1400–2500 nm spectral range.

	**kNN**			**DT**			**RF**		
Class	Prec.	Recall	F-score	Prec.	Recall	F-score	Prec.	Recall	F-score
**Flour type**									
Wheat	0.91	0.91	0.91	0.91	0.91	0.91	0.91	0.91	0.91
Lupin	1.00	1.00	1.00	0.90	1.00	0.95	0.98	0.98	0.98
Chickpea	0.92	0.92	0.92	0.91	0.83	0.87	0.92	0.92	0.92
Grape seed	0.98	0.98	0.98	1.00	1.00	1.00	0.98	0.98	0.98
Olive stone	0.98	0.98	0.98	1.00	1.00	1.00	0.98	0.98	0.98
**accuracy**			**0.96**			**0.95**			**0.95**

**Temperature**									
25	0.96	0.96	0.96	0.85	0.85	0.85	1.00	0.92	0.96
74	0.88	0.91	0.90	0.69	0.85	0.76	0.85	0.85	0.85
110	1.00	0.62	0.76	1.00	0.62	0.76	0.77	0.77	0.77
145	0.70	0.88	0.78	0.54	0.88	0.67	0.58	0.88	0.70
180	0.63	0.71	0.66	0.80	0.62	0.70	0.80	0.62	0.70
**accuracy**			**0.79**			**0.75**			**0.80**
**Phenolics**									
Low	0.99	0.99	0.99	0.99	0.99	0.99	0.99	0.99	0.99
Medium	0.95	0.94	0.95	0.94	0.96	0.95	0.94	0.98	0.95
High	0.99	0.98	0.98	0.96	0.98	0.97	0.99	0.98	0.98
**accuracy**			**0.97**			**0.97**			**0.98**
**Best model and optimal hyperparameters**									
	**Ref.+SG2**<br>**k=7, dist.=Cosine**			**Ref.+SG2**<br>**Max feat.=** $\sqrt{M}$			**Ref.+SG2**<br>**Max. feat.=** $\sqrt{M}$, est.=100		
